# Interventricular septal hematoma detected by transesophageal echocardiography after congenital heart surgery in an infant: a case report

**DOI:** 10.1186/s40001-021-00552-4

**Published:** 2021-08-25

**Authors:** Young-Eun Jang, Jin-Tae Kim, Ji-Hyun Lee

**Affiliations:** Department of Anesthesiology and Pain Medicine, Seoul National University Hospital, Seoul National University College of Medicine, #101 Daehakno, Jongnogu, Seoul, 110-744 Republic of Korea

**Keywords:** Cardiopulmonary bypass, Echocardiography,, Transesophageal echocardiography, Hematoma, Pediatric, Ventricular septal defect, Ventricular septum

## Abstract

**Background:**

Interventricular septal hematoma is an extremely rare complication following congenital heart surgery. During cardiac surgery, interventricular septal hematomas can be detected only by intraoperative transesophageal echocardiography. Here, we report an interesting case of interventricular septal hematoma that was accidentally found in an infant following ventricular septal defect (VSD) closure.

**Case presentation:**

Transesophageal echocardiography images were acquired from a 1-month-old boy after surgical repair of a large (6.5 mm) perimembranous outlet VSD with interventricular septal flattening. Surgical correction was performed with auto-pericardium and 7–0 Prolene sutures. The patient was successfully weaned from cardiopulmonary bypass, and transesophageal echocardiography showed no VSD leakage and good ventricular function. However, approximately 30 min later, two anechoic masses were found within the interventricular septum, which were suspected to be interventricular septal hematomas; the larger mass measured 1.51 $\times$ 1.48 cm. The swollen interventricular septum showed decreased contractility and compressed both the right and left ventricles. However, there was no change in the size of hematomas or a significant hemodynamic instability for 30 min of observation. Therefore, expecting spontaneous resolution of the hematomas, the interventricular septum was not explored, and the patient was removed from cardiopulmonary bypass. On postoperative day 4, follow-up transthoracic echocardiography revealed thrombi filling the hematomas. The patient was discharged on postoperative day 15 and followed up with regular echocardiographic evaluations.

**Conclusions:**

We describe a unique case of interventricular septal hematoma after VSD closure. Surgical manipulation of perimembranous VSD and injury of the septal perforating artery may contribute to the development of an interventricular septal hematoma. Moreover, conservative treatment and serial echocardiographic evaluation generally show gradual hematoma resolution in hemodynamically stable patients. Pediatric cardiac anesthesiologists should be aware of this rare complication after VSD repair.

**Supplementary Information:**

The online version contains supplementary material available at 10.1186/s40001-021-00552-4.

## Background

A ventricular septal defect (VSD) is the most common congenital heart disease [1]. The reported incidence of complications after VSD repair is low; nonetheless, various complications such as heart block, chylothorax, wound infection, or seizures can still occur [2]. Interventricular septal hematoma is an extremely rare complication after congenital heart surgery, which can be life-threatening [3, 4]. In adults, interventricular septal hematoma is associated with a high mortality rate of more than 80% [5]. In contrast, the reported survival rate of pediatric patients is higher than that of adults [3].

We report a case of interventricular septal hematomas that were accidentally detected on transesophageal echocardiography (TEE) after surgical correction of VSD in an infant.

## Case presentation

A 1-month-old boy weighing 3.1 kg was admitted to repair a VSD and atrial septal defect (ASD). The patient was born at 34 weeks, weighing 1.7 kg, and was admitted to the neonatal intensive care unit (ICU). There were no congenital anomalies found except large VSD and ASD in the routine neonatal check. Clinicians decided to perform correction surgery after the patient gained weight. Preoperative medication included diuretics, and there was no abnormal finding in laboratory data, chest radiography, and brain sonography.

Before surgery, transthoracic echocardiography revealed a huge (8 mm) perimembranous VSD with trabecular and outlet extensions, and a 2.5-mm secundum ASD. A bidirectional shunt was found through the VSD with interventricular septal flattening and right ventricular enlargement, attesting to severe pulmonary hypertension.

Surgical correction was performed with cardiopulmonary bypass (CPB) at moderate hypothermia (30’C) after 100U/kg of heparin was administered. Activated clotting time was maintained above 450 s throughout the CPB. The VSD was closed with glutaraldehyde-fixed auto-pericardium and 7-0 Prolene sutures through a transatrial approach. The total CPB time and aorta cross-clamp time were 118 and 68 min, respectively, and the patient was successfully weaned from cardiopulmonary bypass under inotropic support, including dopamine and milrinone. Post-CPB TEE revealed no VSD leakage and good ventricular function with mild interventricular septal flattening.

**Fig. 1:**
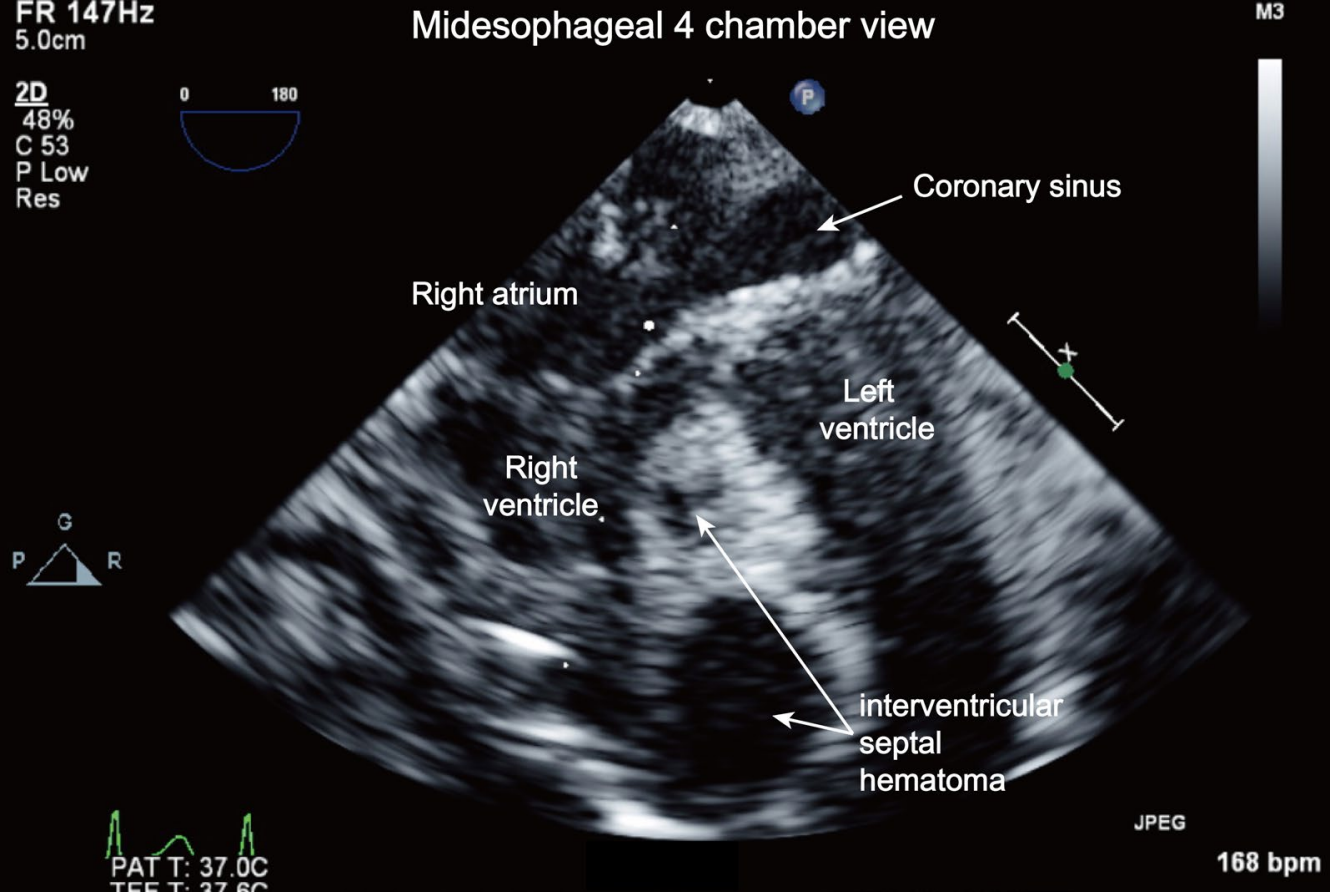
Identification of the anechoic interventricular septal hematomas using the mid-esophageal 4-chamber view (Additional file 1: Video SS1)

**Fig. 2:**
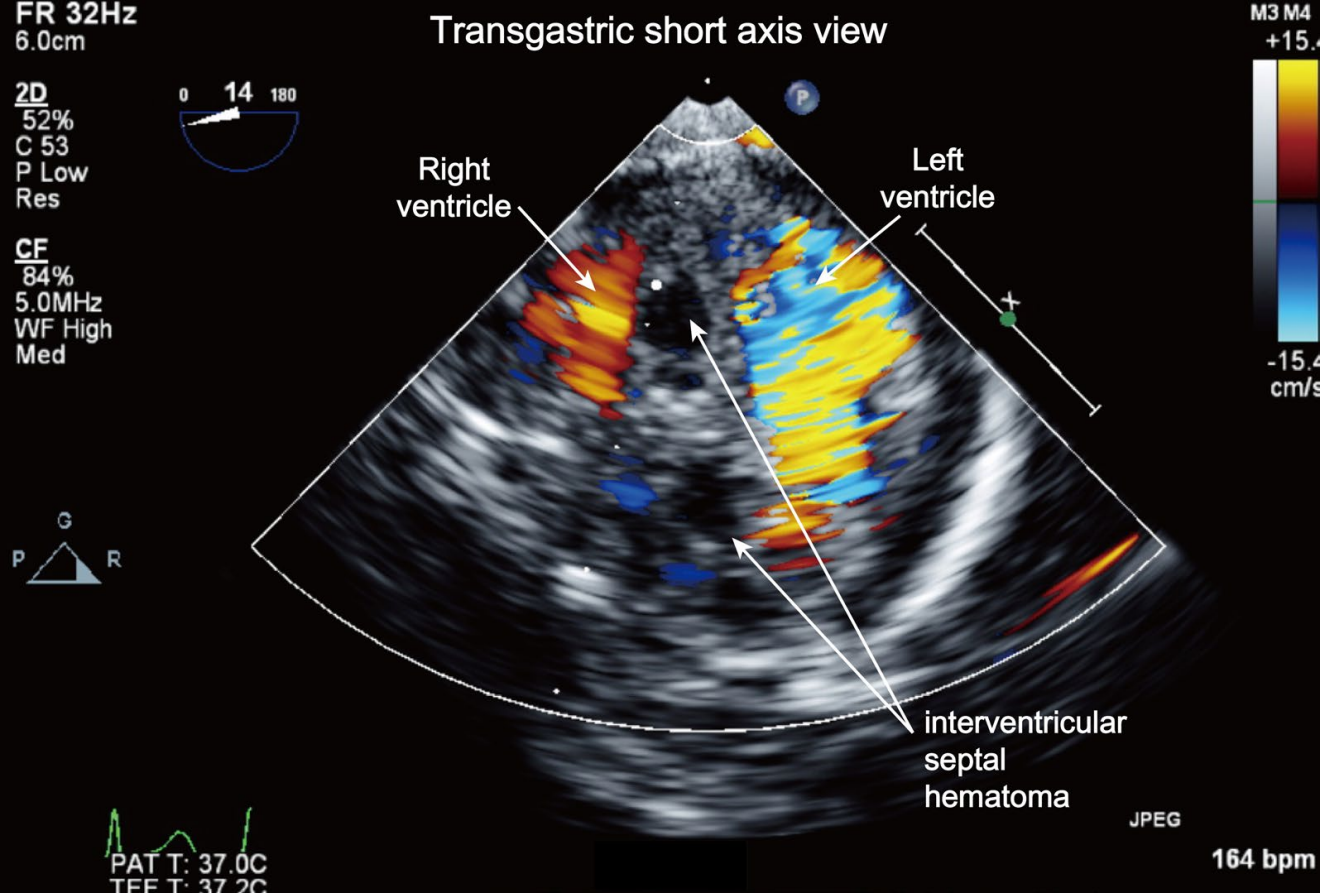
Assessing the connection between the interventricular septal hematomas and the coronary artery using color Doppler images with low Nyquist velocity limits in the transgastric short-axis view. (Additional file 2: Video SS2)

**Fig. 3:**
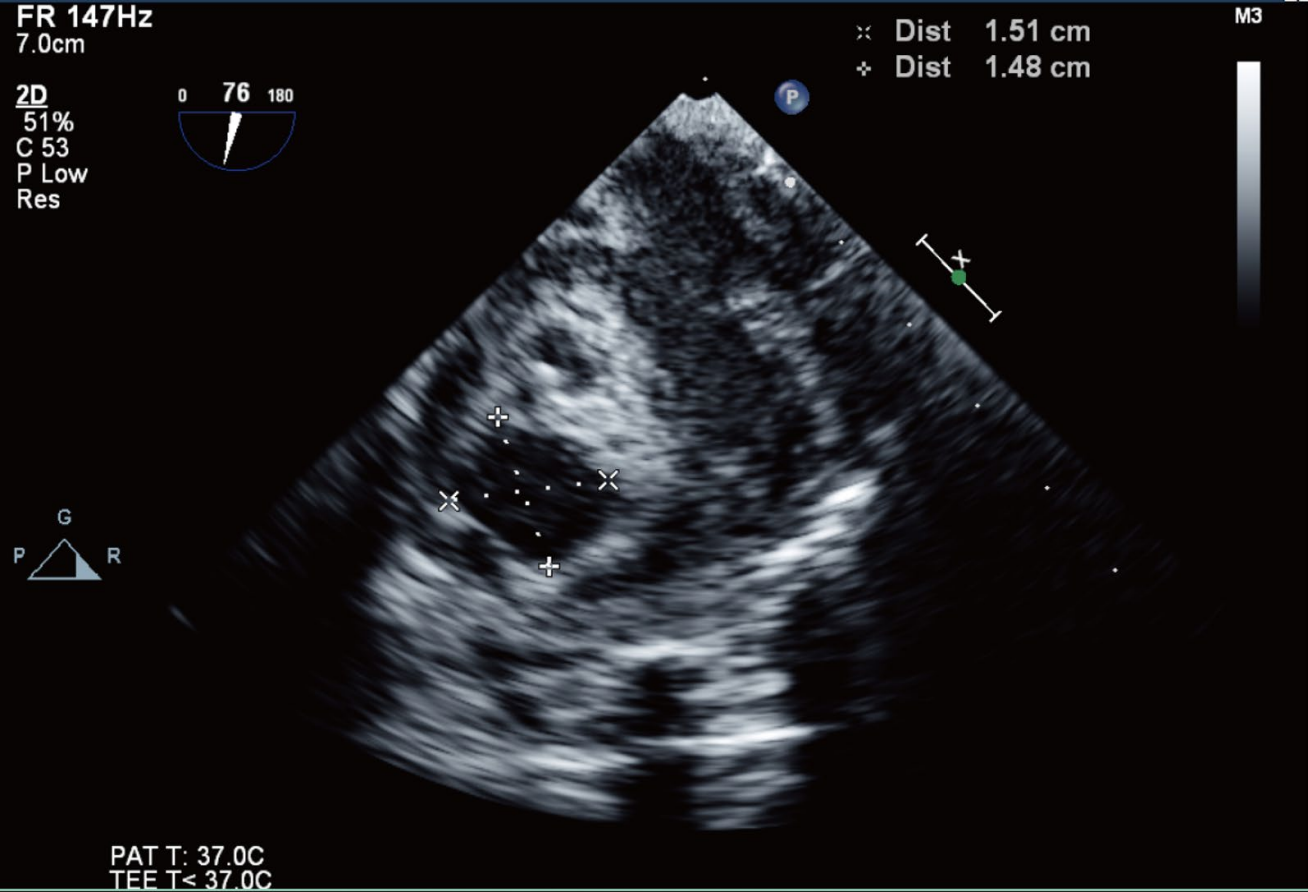
The larger interventricular septal hematoma measuring 1.51 × 1.48 cm

However, approximately 40 min later, two anechoic masses were found within the interventricular septum (Fig. 1, Additional file 1: Video S1). Low‐velocity flow color Doppler echocardiography showed no communication between the ventricles and anechoic masses (Fig. 2, Additional file 2: Video S2). The larger mass was 1.51 $\times$ 1.48 cm, and interventricular septal hematomas were suspected (Fig. 3). The attending surgeon and pediatric cardiologist were immediately notified of these echocardiography findings. The swollen interventricular septum compressed both the right and left ventricles. However, there were no significant mitral stenosis, tricuspid stenosis, and both right and left ventricular outflow tract obstruction. As there was no hemodynamic instability such as arrhythmia or hypotension, and the sternum was already closed, the surgeon decided to observe the patient. The size of hematomas and vital signs did not show any change during 30 min of observation. Therefore, expecting spontaneous resolution of the hematomas, a decision was made not to perform surgical exploration and incisional drainage. The patient was transferred to the ICU, and no specific treatment for hematoma was provided.

**Fig. 4:**
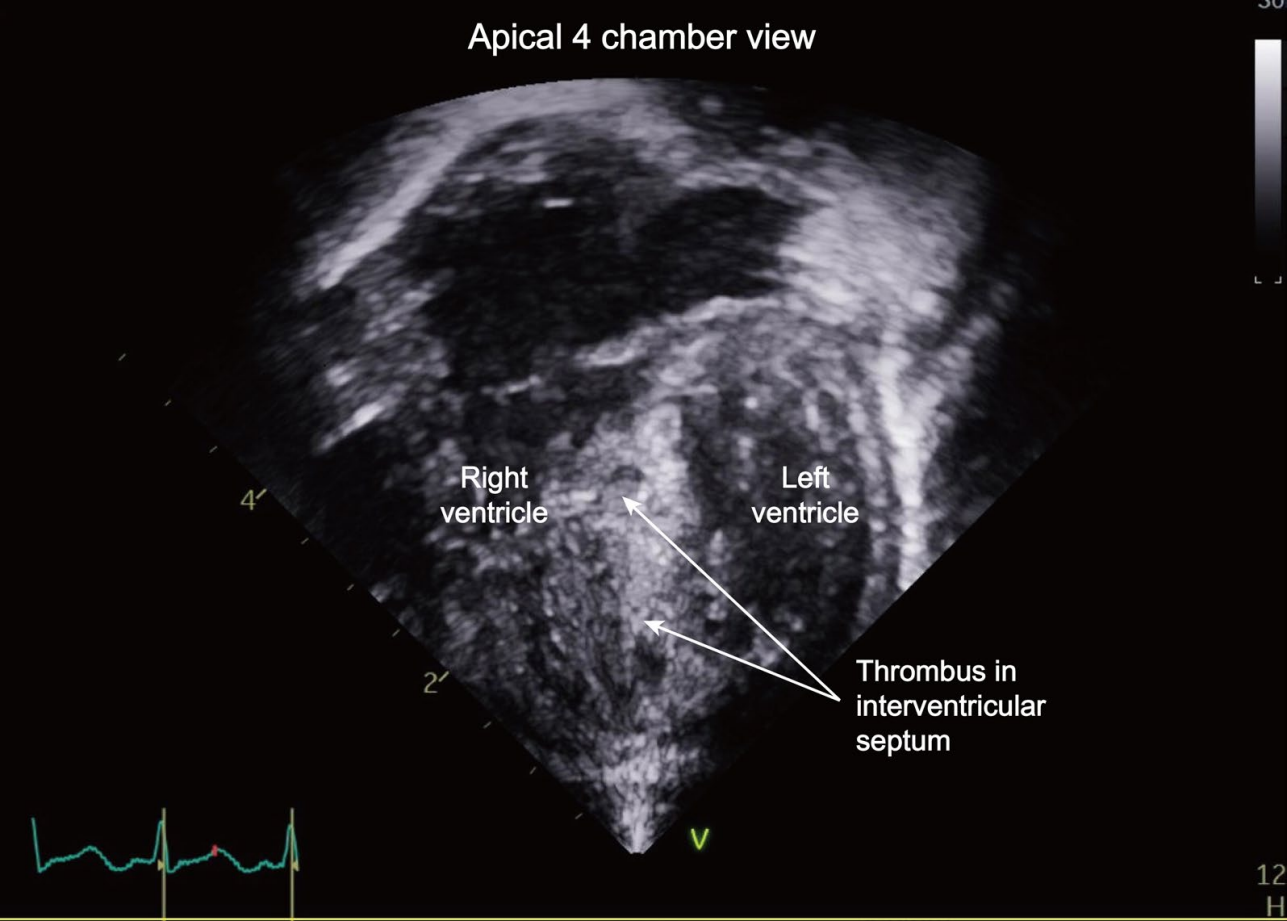
Follow-up transthoracic echocardiography showing a decrease in the size of the anechoic interventricular septal hematomas with thrombi filling the hematomas in the apical 4-chamber view on postoperative day 4. (Additional file 3: Video S3)

**Fig. 5:**
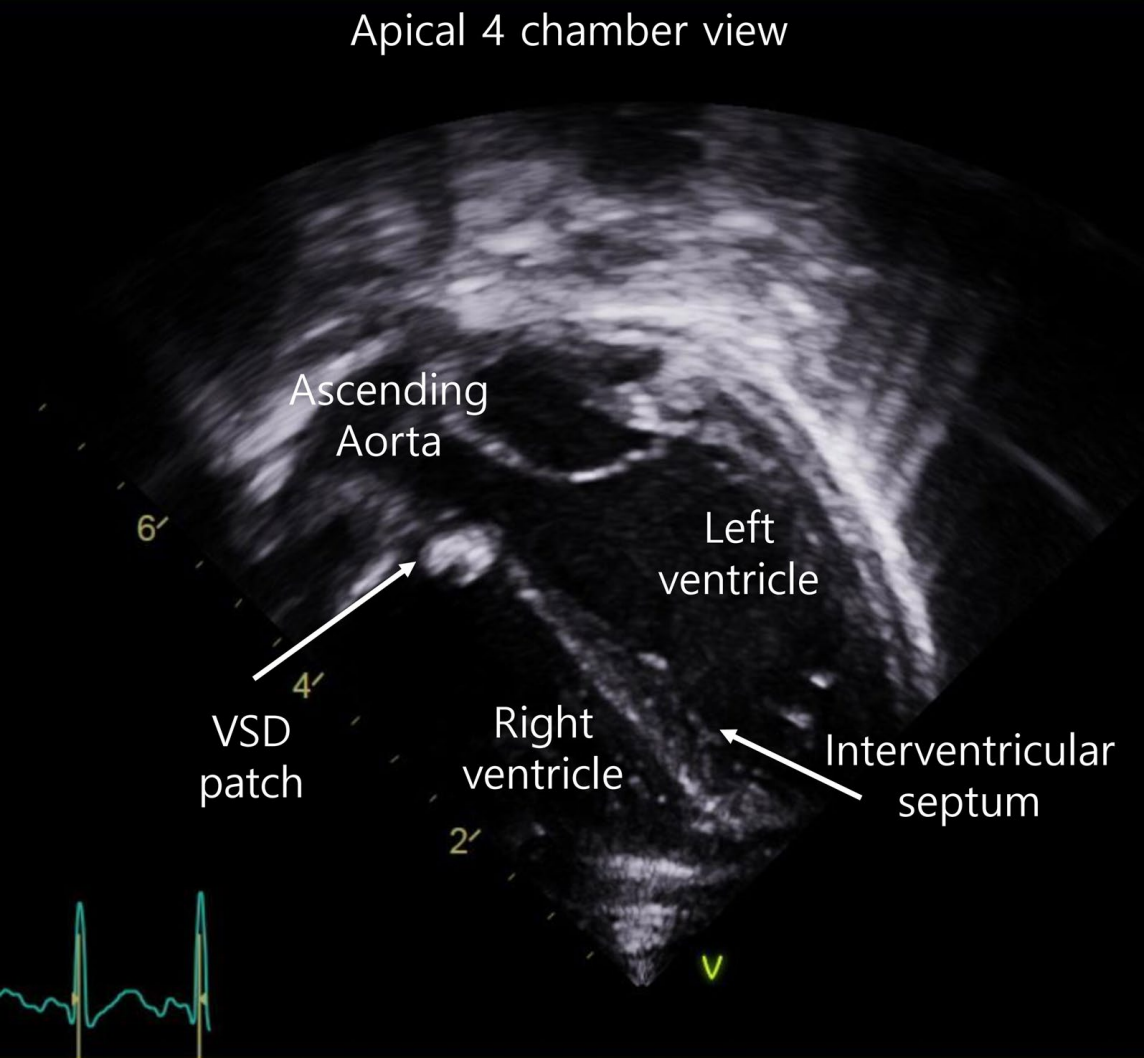
Follow-up transthoracic echocardiography showing a complete resolution of the interventricular septal hematomas in the apical 4-chamber view on postoperative day 40. (Additional file 4: Video S4)

On postoperative day (POD) 4, follow-up transthoracic echocardiography revealed that thrombi were filling the hematomas (Fig. 4, Additional file 3: Video S3). Both ventricular functions were good, with an ejection fraction of 61%, and the interventricular septum thickness was reduced by 5.5 mm. There was no significant hemodynamic instability during the ICU stay. Follow-up echocardiography on POD 14 revealed cystic lesions in the apical ventricular septum, and on POD 40 (Fig. 5, Additional file 4: Video S4), there was no lesion in the interventricular septum, indicating complete resolution of the hematomas.

## Discussion and conclusions

This case report described a large interventricular septal hematoma that was newly detected using TEE after VSD repair in an infant. The patient was transferred to the ICU without surgical intervention, and follow-up echocardiography showed that all hematomas were absorbed without residual intramural lesions.

**Fig. 6:**
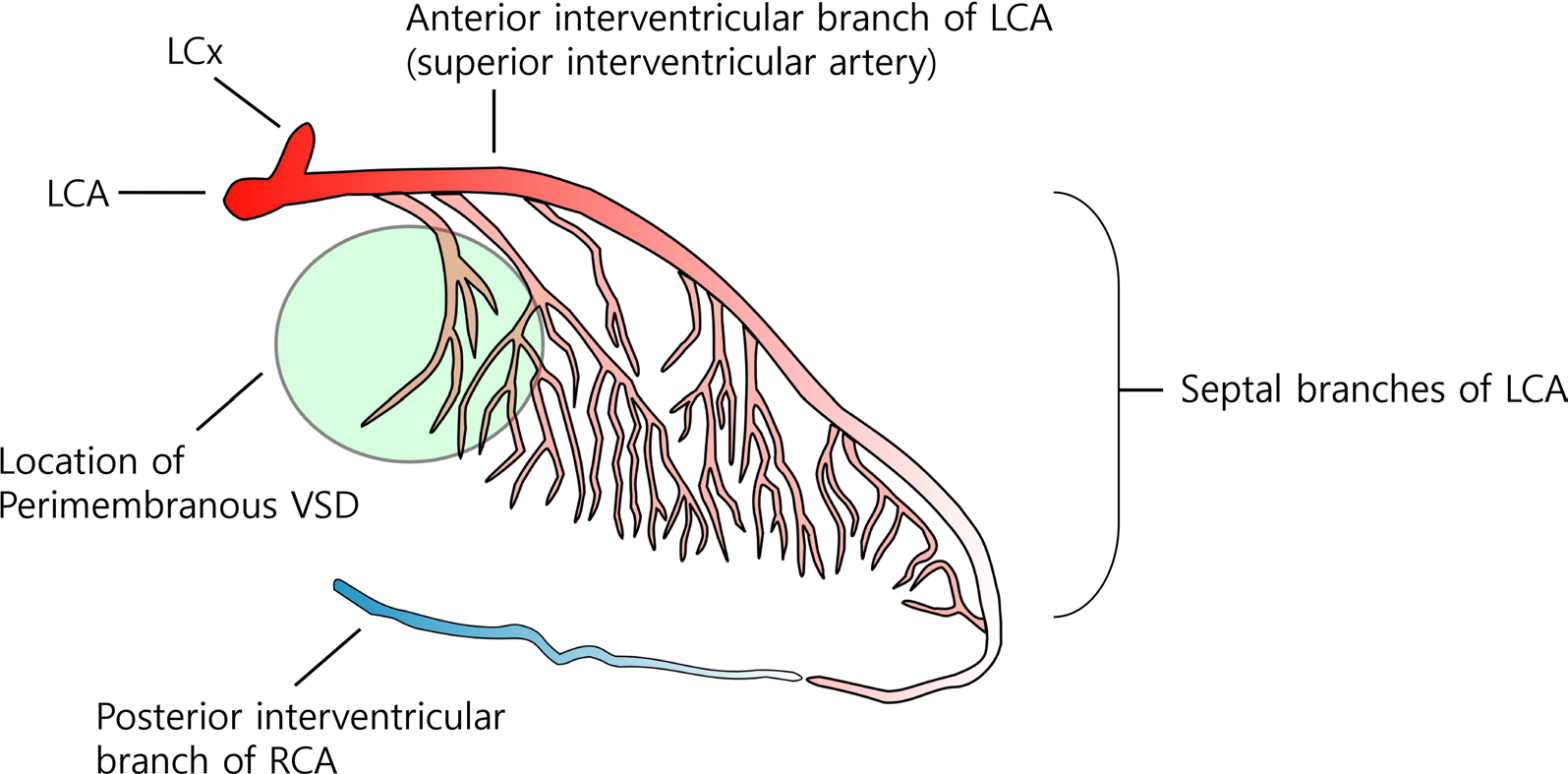
Anatomical considerations during perimembranous ventricular septal defect repair suturing to avoid injuring the interventricular branch from the left coronary artery. The first and second anterior interventricular branches of the left coronary artery could be injured during the operation, and interventricular hematoma can occur

Most cases of interventricular septal hematomas occurred after the closure of perimembranous VSD [4], as in this present case. Surgical injury of the septal perforating artery during VSD closure is suggested to be a contributing factor for the development of ventricular septal hemorrhage [4, 6]. The septal perforating branch passes toward the base of the medial papillary muscle and outlet septum of the right ventricle (RV) from the superior interventricular artery. In a perimembranous VSD, the septal perforating arteries are near the anterior–superior margin of VSD [7, 8] (Fig. 6). Although there might be some variations in coronary arteries, the surgeon should be careful not to place the suture deep in the myocardium, particularly during the closure of the anterior margin of VSD, to prevent injury of the first or the second septal perforating branch.

Surgical trauma and other factors can contribute to the development of intramural hematomas [3]. High-perfusion pressure during cardioplegia and high preoperative RV pressure are other risk factors for myocardial hemorrhages [3, 9, 10]. Suteu et al. reported a case of spontaneous interventricular septal hematoma in an infant with pulmonary atresia with an intact ventricular septum following RV outflow tract reconstruction [3]. The severely increased RV pressure might lead to impaired RV perfusion, development of a myocardial lesion, and intramural hematoma after CPB weaning [3].

Regardless of the cause, the small hematoma due to disruption of the coronary circulation in patients anticoagulated for CPB has the potential to enlarge [11]. A large myocardial hematoma could be associated with decreased ventricular function, myocardial ischemia, or conduction abnormalities, leading to lethal arrhythmias [4, 12, 13]. Some cases of intraventricular septal hematoma showed hemodynamic instability with tachycardia, arrhythmia, ST changes in electrocardiogram [4, 7], or both ventricular outflow tract obstruction [13]. Treatment of myocardial hematoma should be determined based on several factors such as hemodynamic instability, cause and extent of hematoma, or speed of progression [14]. Prompt surgical drainage is required when there is hemodynamic instability or a mechanical complication such as a myocardial defect [14, 15].

In adult patients, intramyocardial hematoma has been described following myocardial infarction or cardiac catheterization [14, 16, 17]. A previous review article demonstrated that intramyocardial hematoma following myocardial infarction could be successfully treated by surgical intervention, while only 10% of patients who received medical treatment survived [15]. In contrast, the reported treatment for interventricular septal hematoma is different in pediatric patients. According to a case report by Drago et al., hemodynamic instability with global septal kinesia and left ventricular outflow tract obstruction occurred due to huge septal hematoma just below the VSD patch in an infant, and prompt surgical exploration should be required [13]. Meanwhile, according to a review article, about half of pediatric patients received conservative management for hematoma after VSD patch closure [4]. Surgical treatment is not required in stable hemodynamics [4, 7] as surgical removal can be associated with the additional myocardial injury and a consecutive loss of functional myocardium [14]. In addition, recurrent hematoma after drainage was reported [11]. Some clinicians used antithrombotic agents for conservative therapy, especially when there was evidence of myocardial infarction or aneurysmal changes in the myocardium [7]. In this case, the hematoma was gradually filled with thrombi, and septal thickness gradually decreased at the immediate postoperative period, without functional abnormalities in echo data. Therefore, no further treatment was necessary.

During congenital heart surgery, an interventricular septal hematoma can be detected by intraoperative TEE [4, 11, 18]. Although most similar cases were detected immediately after CPB, our case was detected 30 min after weaning from CPB. Therefore, pediatric cardiac anesthesiologists should perform vigilant monitoring using TEE, which can facilitate the early detection of complications following congenital heart surgery [12, 19].

Regular follow-up echocardiography is recommended to confirm the complete resolution of hematomas or detect delayed complications, including newly developed aneurysms, myocardial infarction, arrhythmia, or myocardial rupture. Yamazawa et al. recommended follow-up by echocardiography every 2–3 months within 6 months after the detection of an interventricular septal hematoma [7].

In conclusion, the present case suggests that interventricular septal hematoma can occur after VSD closure, although its incidence is extremely low. Continuous TEE monitoring during anesthesia is essential to detect this rare complication immediately after congenital heart surgery. Close follow-up using echocardiography is recommended for several weeks to monitor absorption of the hematoma.

## Supplementary Information

**Additional file 1: Video S1.**

**Additional file 2: Video S2.**

**Additional file 3: Video S3.**

**Additional file 4: Video S4.**

## Data Availability

Data sharing is not applicable to this article as no datasets were generated or analyzed during the current study.

## Abbreviations

ASD: Atrial septal defect
CPB: Cardiopulmonary bypass
ICU: Intensive care unit
POD: Postoperative day
RV: Right ventricle;
TEE: Transesophageal echocardiography
VSD: Ventricular septal defect
